# Silk peptide treatment can improve the exercise performance of mice

**DOI:** 10.1186/1550-2783-11-35

**Published:** 2014-07-01

**Authors:** Jisu Kim, Hyejung Hwang, Jonghoon Park, Hea-Yeon Yun, Heajung Suh, Kiwon Lim

**Affiliations:** 1 Laboratory of Exercise Nutrition, Department of Physical Education, Konkuk University, 120, Neungdong-ro, Gwangin-gu, Seoul 143-701, Republic of Korea

**Keywords:** Silk peptide, Exercise performance, Energy metabolism during exercise, Fat oxidation

## Abstract

**Background:**

We previously reported that silk peptide (SP) treatment led to increased resting fat oxidation in exercised mice. However, it was not known whether SP treatment could effectively increase exercise capacity. Accordingly, this study aimed to examine whether SP treatment affected energy metabolism during exercise in addition to exercise performance.

**Methods:**

We randomized 36 7-week-old male ICR mice into 2 groups: the control (n = 18) and SP (n = 18) groups. All mice were trained by treadmill running 5 times per week for 2 weeks. SP was dissolved in distilled water and daily 800-mg/kg body weight doses before the running exercise were oral administered intraperitoneally to the SP group for 2 weeks. $\dot{V}O_{2}\max$ was measured before and after the 2 weeks training period. We also assessed energy metabolism during exercise for 1 h after the 2 week training period. In addition to blood samples, liver glycogen and gastrocnemius-white and gastrocnemius-red muscle was obtained at the following 3 time points: at rest, immediately after exercise, and 1-hour post exercise.

**Results:**

The $\dot{V}O_{2}$ max after 2 weeks of training was significantly increased (8%) in the SP group compared to the baseline; a similar result was not observed in the CON group. The sum of fat oxidation during a 1-h period tended to be 13% higher in the SP group than in the CON group (*P* < 0.077). In particular, the sum of fat oxidation was significantly higher in the SP group during the initial 20-min phase than that in the CON group (*P* < 0.05). The glycogen concentration in the white gastrocnemius muscle did not differ between the groups either rest or after 1 h of exercise but was significantly higher in the SP group than in the CON group during the recovery period (1 h post-exercise completion).

**Conclusions:**

These results suggest that SP treatment can improve the exercise performance. Therefore, SP is considered to confer beneficial effects upon athletes, in whom exercise abilities are required.

## Background

The 3 key factors of athletic performance enhancement are training, nutrition, and rest [1]. Of these, the diet chosen by an athlete will affect his performance on and off the track through its effects on both fitness and health [2]. Therefore, many athletes have used dietary supplements to increase their exercise capacities [3-5]. However, many of these dietary supplements have added artificial chemical and overdoses have caused many side effects [6,7]. As a result, many researchers have been investigating natural ergogenic foods that do not cause any side effects.

Silk peptide (SP) has been ingested for many years in Asian countries [8]. SP comprises biopolymers from the cocoons produced by silkworms for protection from the environment during metamorphosis to the mature moth stage [8]. SP is a natural biomolecule used in powder and extract forms in diverse pharmacological capacities as well as in biomedical and biotechnological fields [9-11]. Recently, studies have reported the benefits of SP treatment on endurance exercise in rodent models [12,13]. Shin *et al*. [12] demonstrated that in mice, SP improved physical stamina in a dose-dependent manner during a maximum swimming exercise. The authors also reported that SP exhibited stamina-enhancing and anti-fatigue activities in mice during forced swimming by preventing tissue (liver and muscle) injuries and glycogen-sparing effects [13]. Moreover, SP was found to reduce blood circulation to injured muscles and liver tissues while increasing the numbers of red blood cells [14]. However, to our knowledge, the effects of SP treatment on energy metabolism alterations during exercise and $\dot{V}O_{2}$ max improvements have not been examined.

We previously reported that SP treatment could increase resting fat oxidation in exercised mice [15]. Therefore, we hypothesized that SP treatment could also improve the exercise performance along with increasing the fat oxidation during exercise. Accordingly, the purpose of this study was to evaluate the effects of SP treatment on endurance exercise performance and energy metabolism during running exercise, using a respiratory open-circuit system for rodents.

## Methods

### Animals and protocol

Seven-week-old male ICR mice (n = 36) were used. The mice were purchased from Orient Bio, Inc. (Seongnam, Korea). Initially, the mice were randomized into 2 groups, the control group (CON; distilled water with training, n =18) and the SP treatment group (SP; SP-treated with training, n = 18). All mice were trained by treadmill running 5 times per week for 2 weeks. SP was dissolved in distilled water and 800-mg/kg body weight daily doses and administered orally intraperitoneally before the running exercise to the SP group for 2 weeks [13-15]. The CON group was treated with vehicle only (distilled water 5 mL/kg body weight). $\dot{V}O_{2}\max$ was measured before and after the 2 weeks training period. We also evaluated energy metabolism during exercise for 1 h after the 2 weeks training period. Mice were fasted 3 h before the 1 h exercise. We obtained blood, liver glycogen, and gastrocnemius-white and red muscle samples at three time points: rest, immediately after exercise and 1 h post-exercise. The mice were fed ad libitum with a standard diet (5 L79; Orient Bio, Inc.) containing the following nutrients (g/kg diet): crude protein, 180; crude fat, 52; crude fiber, 52; minerals, 57; and carbohydrates, 368. The calorically based protein:fat:carbohydrate ratio (%) was 21:14:65, and the gross and metabolizable caloric contents of the diet were 4.04 and 3.21 Kcal/g, respectively. The body weights and food intake were monitored daily throughout the experiment. All mice were housed in standard plastic cages under controlled humidity (50%) and temperature (23°C ± 1°C) conditions and with alternating 12-h light/dark cycles. All experimental procedures were performed at the Animal Experiment Research Center of Konkuk University. This study was conducted in accordance with the ethical guidelines of the Konkuk University Institutional Animal Care and Use Committee.

### Silk peptides

SP were obtained from Worldway Co. Ltd. (Jeoneui, Korea). The SP primarily comprised amino acids in the following order of concentration: Ala (34.36%) > Gly (27.23%) > Iso (15.51%) > Ser (9.58%) > minor amino acids. Composition details are shown in Table 1. The SP composition according to molecular weight was as follows: an approximate range of 150–350 D and an average molecular weight of approximately 250 D.

**Table 1:** Amino acid compositions (%) of SP

**Amino acid**	**SP (silk peptide)**
Ala	34.36
Gly	27.23
Iso	15.51
Ser	9.58
Val	3.49
Thr	2.00
Asp	1.68
Glu	1.28
Ile	1.25
Leu	1.24
Phe	0.87
Pro	0.44
Tyr	0.41
His	0.21
Arg	0.17
Met	0.10
Lys	0.10
Cys	0.05
Trp	0.05
Sum	100.00

### Training method

Running mice were adapted to treadmill training (treadmill from Daejong Systems, Korea) at a fixed intensity (15 m/min, 8° slope) for 3 days. All mice were then tested for a certain period at a frequency of 5 times per week for a total of 2 weeks. The following protocols were used: 20 m/min, 8° slope, 50 min/day for the first week and 25 m/min, 8° slope, 50 min/day (about 75% of maximum $\dot{V}O_{2}$) for the second week [16].

### Maximal oxygen uptake $\left(\dot{V}O_{2}\max\right)$ and energy metabolism during exercise

The $\dot{V}O_{2}$ max test was performed before and after the experimental period, using an open circuit calorimetry system. The maximal oxygen uptake protocol was used in accordance with previous studies [16,17]. Briefly, the initial slope and speed were set at 0° and 14 m/min, respectively, and were then increased by 2° and 2 m/min, respectively, every 2 min; the mice were measured in the same environment both before and after training. After 2 weeks of training, the energy metabolism during exercise was measured at the same training intensity as during the second week (25 m/min, slope of 8°, 75% of maximum $\dot{V}O_{2}$) for 1 h. The mice were placed in exercise metabolism chambers for adaptation at 2 h before the measurement [16].

### Gas analysis

Respiratory gas was measured with an open-circuit apparatus in accordance with previous studies [15,16,18]. The O_2_ uptake and CO_2_ production were measured with a mass analyzer (gas analyzer model RL-600; Alco System, Chiba, Japan) and a switching system (model ANI6-A-S; Alco System). The flow rate was maintained at 3 L/min. The O_2_ uptake and CO_2_ production were used to calculate the RER, carbohydrate oxidation, and fat oxidation in the mice.

### Glycogen analysis

Glycogen contents in the muscles and liver were measured in a perchloric acid extract according to the amyloglucosidase method [19].

### Blood analysis

Blood samples were collected rest, immediately after exercise and 1 h post-exercise. Plasma glucose was measured using commercial kits (Asan Pharmaceutical Co., Hwaseong-si Gyeonggi-do, Korea), the plasma FFA level using a non-esterified fatty acid kit (Wako Pure Chemical Industries), and the plasma insulin level was determined with an enzyme-linked immunosorbent assay kit (Morinaga Bioscience Laboratory, Yokohama, Japan).

### Statistical analysis

All data are presented as means ± standard deviations (SD). All statistical analyses were performed with SPSS version 19.0 software (SPSS, Inc., Chicago, IL, USA). Differences between the groups were analyzed with an unpaired *t*-test. The one-way analysis of variance was used to determine the changes in $\dot{V}O_{2}$ max before and after training, blood analysis and the changes in glycogen contents during and at 1 h after exercise in the CON and SP groups. A Bonferroni post-hoc analysis was conducted if significance was obtained. The changes in fat oxidation on energy metabolism during exercise were analyzed with a two-way repeated measures analysis of variance. Statistical significance was defined as *P* < 0.05.

## Results

### Body weights, food consumption, and adipose tissue weights in the CON and SP groups

The body weights, food consumption, and adipose tissue weights are shown in Table 2. The final body weights and body weight gains were significantly lower in the SP group than in the CON group. The food consumption was significantly higher in the SP group than in the CON group. The total weights of the abdominal adipose tissue and epididymal tissue were significantly lower in the SP group than in the CON group.

**Table 2:** The change of body weight, food intake and adipose tissue weight in CON and SP groups

**Initial body weight (g)**	**34.7±0.6**	**34.3±0.3**
Final body weight (g)	37.1 ± 2.1	35.7 ± 1.3*
Body weight gain (g)	2.8 ± 1.4	1.4 ± 1.0**
Food intake (g/day)	4.4 ± 0.3	4.9 ± 0.3***
Food efficiency ratio	0.7 ± 0.2	0.3 ± 0.0***
Abdominal tissue (g)		
Epididymal	0.48 ± 0.0	0.42 ± 0.10*
Perirenal	0.15 ± 0.0	0.12 ± 0.04
Mesenteric	0.51 ± 0.0	0.48 ± 0.07
Total adipose tissue	1.15 ± 0.1	1.03 ± 0.17*

The change of body weight, food intake and adipose tissue weight. CON: untreated with training, SP: silk peptide-treated with training. Values are presented as means ± standard deviations (n=36). Significant difference between groups are indicated by *p<0.05, **p<0.01, ***p<0.001.

### Effect of the maximal oxygen uptake $\left(\dot{V}O_{2}\max\right)$

**Figure 1:**
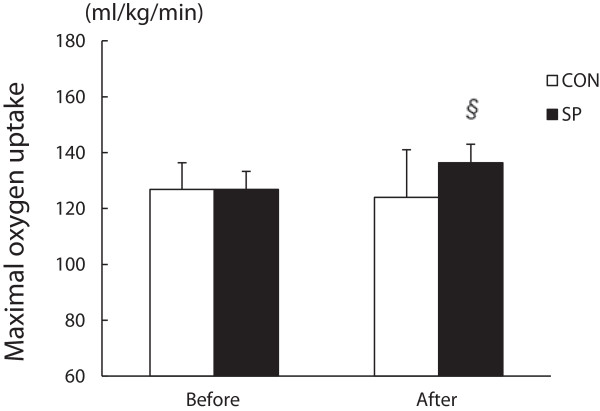
**Change in the maximal oxygen uptake**$\left(\dot{V}O_{2}\max\right)$**before and after training.** CON: distilled water with training, SP: silk peptide-treated with training. Values are presented as means ± standard deviations (n = 12). § vs. Before, *P* < 0.05.

In the SP group, the $\left(\dot{V}O_{2}\max\right)$ after 2 weeks of training increased significantly (8%) when compared with that observed before training (before, 126.8 ± 6.4 mL/kg/min; after, 136.3 ± 6.6 mL/kg/min); a similar result was not observed in the CON group (Figure 1).

### Energy metabolism alterations during exercise

**Figure 2:**
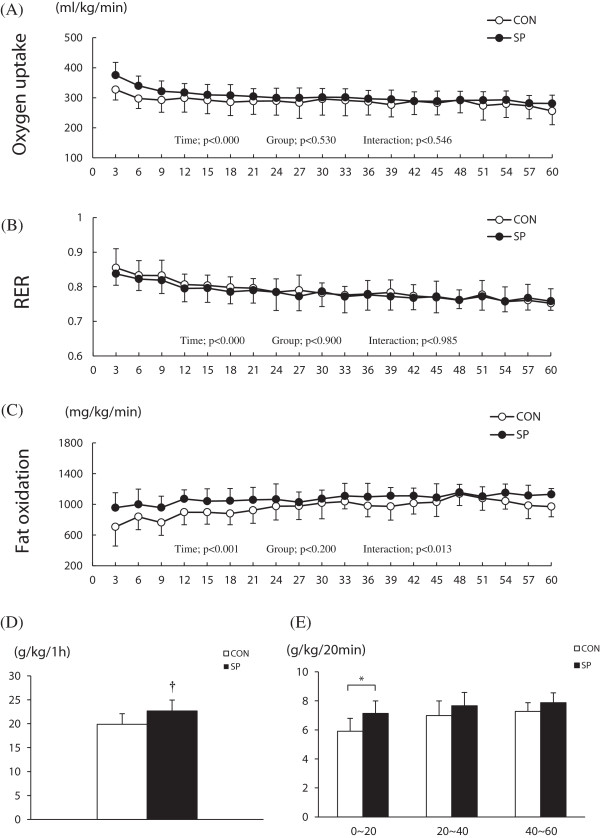
**Change in the oxygen uptake, RER and fat oxidation level during a 1-h exercise period.** CON: distilled water with training, SP: silk peptide-treated with training. **A**, the change in oxygen uptake over a 1-h period; **B**, the change in RER over a 1-h period; **C**, the change in fat oxidation over a 1-h period; **D**, the sum of the fat oxidation over a 1-h period; **E**, fat oxidation during the 20-min period. Values are presented as means ± standard deviations (n = 12). † vs. CON *P* < 0.077; * vs. CON, *P* < 0.05.

The oxygen uptake and RER was shown the time effect, but not different between the groups (Figure 2A,B). Fat oxidation during a 1-h exercise period was calculated from the $\left(\dot{V}CO_{2}\right)$ and $\dot{V}O_{2}$ values, and a significant time effect and an interaction were observed (Figure 2C). The sum of fat oxidation during a 1-h period tended to be 13% higher in the SP group than in the CON group (*P* < 0.077; Figure 2D). In particular, fat oxidation was significantly increased during the initial 20-min phase in the SP group, compared with that in the CON group (*P* < 0.05; Figure 2E).

### Blood analysis

**Figure 3:**
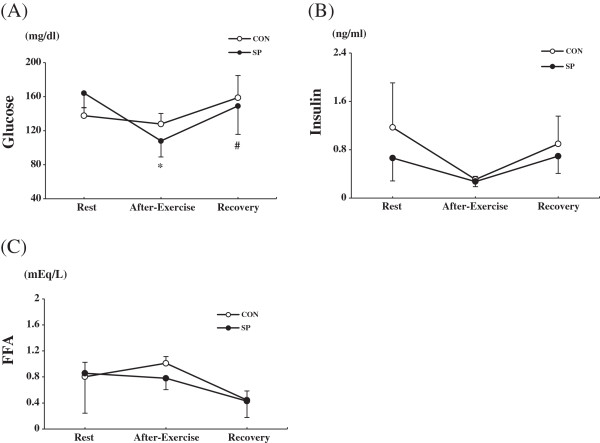
**Changes in the plasma glucose, insulin and FFA levels during exercise and after 1 h of exercise.** CON: distilled water with training, SP: silk peptide-treated with training. **A**, Glucose; **B**, Insulin; **C**, FFA (free fatty acids) at rest, after exercise, and recovery in the CON and SP groups. Values are presented as means ± standard deviation (n = 36). * vs. rest, *P* < 0.001; # vs. After-exercise, *P* < 0.01.

The plasma glucose levels was not significantly different between the groups at any time point. However, The plasma of glucose levels was significantly lower immediately after exercise time point than rest time point in the SP group and this increase was recovered at the 1 h post-exercise (recovery phase) (Figure 3A). The insulin and FFA levels did not differ between the groups at any time point (Figure 3B,C).

### Glycogen concentrations in the tissues

**Figure 4:**
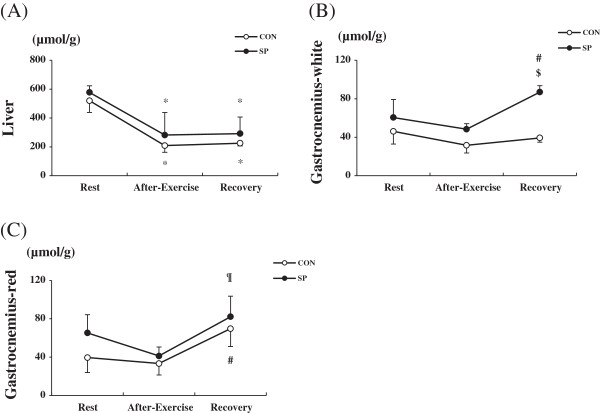
**Changes in the glycogen levels during exercise and after 1 h of exercise.** CON: distilled water with training, SP: silk peptide-treated with training. **A**, liver; **B**, white gastrocnemius muscle tissue; and **C**, red gastrocnemius muscle tissue at rest, after exercise, and recovery in the CON and SP groups. Values are presented as means ± standard deviations (n = 36). * vs. rest, *P* < 0.01; # vs. rest and after-exercise, *P* < 0.05; $ vs. recovery in CON, *P* < 0.001; ¶ vs. after-exercise, *P* < 0.05.

The glycogen concentration in the liver did not differ between the groups at any of the time points (Figure 4A). Furthermore, the glycogen concentration in the white gastrocnemius muscle tissue did not differ between the groups at the rest and immediately post-exercise time points; however, this variable was significantly higher in the SP group than in the CON group at the recovery period time point (1 h post-exercise; Figure 4B). In contrast, no significant between-group differences were observed in the red gastrocnemius muscle tissue (Figure 4C).

## Discussion

The present study demonstrated that a 2-week regimen of silk peptide (SP) treatment and endurance training could increase the $\dot{V}O_{2}$ max, whereas endurance training alone had no similar effect. A 2-week period of SP treatment also increased fat oxidation during the initial phase of exercise in exercised mice.

In human studies, the $\dot{V}O_{2}$ max test during graded treadmill exercise is the most commonly used endurance performance measurement [20,21]. In the present study, $\dot{V}O_{2}$ max was not changed in the CON group after the 2-week training. Our previous study demonstrated that $\dot{V}O_{2}$ max was significantly increased by 4 week-training which the intensity was the same with the present study training protocol [16]. Thus, the duration (2 weeks) and/or intensity (75% of VO_2_ max) seem not to be enough to increase the endurance capacity in the present study. On the other hand, the $\dot{V}O_{2}$ max was significantly increased after a 2-week period of SP treatment when compared with the same metric before training. A previous study reported that a 30-day SP treatment regimen (800 mg/kg body weight daily) and swimming exercise training increased the maximum swimming time of mice by reducing exercise-induced tissue injuries and energy depletion [13]. In addition, a 44-day SP treatment regimen led to an increased maximum swimming time and decrease in the levels of muscle tissue damage markers such as creatine kinase, aspartate aminotransferase, and lactate dehydrogenase in a dose-dependent (50, 160, and 500 mg/kg) manner after forced swimming exercises [12]. Therefore, it seems that SP treatment can increase the exercise capacity regardless of the type of exercise.

We found that the fat oxidation during 1 h of exercise tend to be 13% higher in the SP group than in the CON group; in particular, we found that the initial 20-min fat oxidation phase during the 1-h exercise period was significantly increased in the SP group, compared with that in the CON group. Sun *et al*. [11] assessed the effects of SP on adipogenesis in mature adipocytes in vitro and the effects against obesity in vivo. As a result, an 8-week SP treatment period inhibited both preadipocyte differentiation and adipogenesis and reduced the body and fat weights in induced-obese rats that were fed a high-fat diet. Additionally, Lee *et al*. [22] reported that SP treatment reduced fat accumulation by up-regulating leptin in 3 T3-L1 fibroblasts. We previously reported that SP treatment promoted resting fat oxidation [15]. To our knowledge, the results of the present study provide the first evidence of a further increase in fat oxidation during exercise in mice treated with SP relative to those not treated with SP. Taken together, these data indicate that SP might increase the exercise capacity by modulating fat metabolism during exercise.

The present study demonstrated no significant glycogen-saving effects of a 2-week SP treatment regimen during exercise. However, somewhat surprisingly, the glycogen concentration in the white gastrocnemius muscle tissue increased in the SP group during the recovery period (at 1 h post-exercise). Previous studies have reported that SP treatment for more than 1 month yielded glycogen-saving effects [12,13]; however, these previous studies did not analyze the glycogen levels at the post-exercise recovery time point. The discrepancy between the current and previous studies regarding the glycogen-saving effect might have been due to the SP treatment duration or dose or the different types of exercise to which the animals were subjected. A number of investigators have reported post-exercise increases in the total glycogen synthase activity levels in skeletal muscle tissues [23-25]. Therefore, it appears that increase glycogen synthase activity would exert beneficial effects with SP at 1 h post-exercise. It remains unclear why the 2-week SP treatment used in the present study led to increased post-exercise accumulation. We also found that glucose, FFA and insulin levels in plasma did not differ between the groups. Particularly, the glucose level was significantly decreased at immediately after exercise and increased 1 h post-exercise in the SP group. However, the alteration of the glucose level in SP group seems to be involved with the glycogen synthase in the recovery period. In a future study it will be necessary for us to study the effect of SP on fat and carbohydrate metabolism related to gene expression in detail.

We could not exclude the possibility that higher fat oxidation of SP mice would be due to lower intensity of exercise after 2-wk training but not to a direct effect of SP. We did not use untrained mice as the control because we intended to prove the effects of supplementation of SP in exercise training state. However, to clarify the direct effect of SP and the synergistic effects of SP administration in combination with exercise on energy metabolism more in detail, it would be important to add a resting group to the present experimental setting or to extend the experimental period.

## Conclusions

In conclusion, these results suggest that SP intake can improve exercise performance. Therefore, SP is considered to confer beneficial effects upon athletes, in whom an exercise ability and fat loss are required. It will be necessary to clarify the effect of SP on endurance capacity in trained human athletes and also to understand the mechanism that underlies the effect of SP on fat and carbohydrate metabolism-related gene expression in the skeletal muscles in future studies.

## Competing interests

The authors declare that they have no competing interests.

## Author’ contributions

JK analysed and interpreted the data and wrote the manuscript. HH and HY analysed data. JP interpreted the data and wrote the manuscript. KL interpreted the data and had primary responsibility for the final content. HS interpreted the data. All authors approved the final version of the manuscript.
